# Perceptions of the primary health care team about the implementation of integrated care of patients with type 2 diabetes and hypertension in Slovenia: qualitative study

**DOI:** 10.1186/s12913-023-09353-3

**Published:** 2023-04-12

**Authors:** Nataša Stojnić, Zalika Klemenc-Ketiš, Majda Mori Lukančič, Črt Zavrnik, Antonija Poplas Susič

**Affiliations:** 1 Community Health Centre Ljubljana, Ljubljana, Slovenia; 2 Department of Family Medicine, Faculty of Medicine, University of Ljubljana, Ljubljana, Slovenia; 3 Department of Family Medicine, Faculty of Medicine, University of Maribor, Maribor, Slovenia

**Keywords:** Integrated health care system, type-2-diabetes, Hypertension, Health care team, interdisciplinary primary care, qualitative research

## Abstract

**Background:**

Integrated care involves good coordination, networking, and communication within health care services and externally between providers and patients or informal caregivers. It affects the quality of services, is more cost-effective, and contributes to greater satisfaction among individuals and providers of integrated care. In our study, we examined the implementation and understanding of integrated care from the perspective of providers - the health care team - and gained insights into the current situation.

**Methods:**

Eight focus groups were conducted with health care teams, involving a total of 48 health care professionals, including family physicians, registered nurses, practice nurses, community nurses, and registered nurses working in a health education center. Prior to conducting the focus groups, a thematic guide was developed based on the literature and contextual knowledge with the main themes of the integrated care package. The analysis was conducted using the NVivo program.

**Results:**

We identified 12 main themes with 49 subthemes. Health care professionals highlighted good accessibility and the method of diagnostic screening integrated with preventive examinations as positive aspects of the current system of integrated care in Slovenia. They mentioned the good cooperation within the team, with the involvement of registered nurses and community nurses being a particular advantage. Complaints were made about the high workload and the lack of workforce. They feel that patients do not take the disease seriously enough and that patients as teachers could be useful.

**Conclusion:**

Primary care teams described the importance of implementing integrated care for diabetes and hypertension patients at four levels: Patient, community, care providers, and state. Primary care teams also recognized the importance of including more professionals from different health care settings on their team.

**Supplementary Information:**

The online version contains supplementary material available at 10.1186/s12913-023-09353-3.

## Background

The term integrated care is often used interchangeably with terms such as coordinated care and seamless care, emphasizing the central role of population and individual needs [1, 2]. A more specific definition of integrated health care might be that it is managed and delivered in such a way that people receive a continuum of health promotion, disease prevention, diagnosis, treatment, disease management, rehabilitation and palliative care that is coordinated across levels and sites of care within and beyond the health sector and meets their needs across the life course [3].

Integrated care can increase patient satisfaction, improve perceived quality of care, and facilitate access to services [4, 5]. It can also reduce hospital admissions and length of hospital stay, lower costs, and improve care outcomes [6, 7].

Integrated care typically involves comprehensive community-based care for people with chronic conditions. Noncommunicable diseases (NCDs) are the leading cause of more than 70% of deaths worldwide and pose a major threat to the economy and society. Diabetes and cardiovascular disease (led by hypertension) are two of the top four noncommunicable diseases that cause the most deaths [8–10]. In Slovenia, noncommunicable diseases are responsible for an estimated 88% of all deaths, including 2% for diabetes and 40% for cardiovascular disease [9, 11]. The key to reducing premature mortality and morbidity is early detection and timely treatment of diseases through a good health care system [12].

According to the recent World Health Organization (WHO) report on integrated primary care in Slovenia [13], Slovenia has a well-functioning primary care system. This could be attributed in part to the successful integration of public health and primary care. This way of working has contributed to an impressive decline in the burden of disease from noncommunicable diseases and a rapid increase in life expectancy at birth [13, 14].

In Slovenia, there are family medicine practices with a family physician (FP), a practice nurse, and a registered nurse. Practice nurse is a secondary school degree nurse whose tasks in the family practice teams are administration, appointments, and clinical work (taking care of wounds, point-to-care measurements etc.) [15]. A registered nurse actively participates in the preventive care (screening) of the target population of patients (i.e., those over 30 years of age) and in the comprehensive management of patients with the most common chronic noncommunicable diseases (including type 2 diabetes and hypertension) as defined by FP. The family medicine practices collaborate with teams at health promotion centres and a community nurse [16, 17]. A registered nurse actively invites patients from a targeted population to the family medicine practice for screening for the most common chronic diseases and provides advice on prevention. Each person is invited for screening every five years. If the person does not respond to the invitation for screening, the FP or registered nurse can contact the community nurse, who can visit the person who did not respond at home [18, 19].

The quality of family medicine practices’ work in Slovenia is continuously monitored using quality indicators [17, 20]. These are mainly clinical indicators for prevention and management of chronic diseases. The quality of integrated care is not measured directly. In addition, there are no data on the views and opinions of primary care practitioners on integrated care in Slovenia. Therefore, the aim of this research was to investigate the views and opinions of primary health care teams on integrated care for patients with type 2 diabetes and hypertension in primary care.

## Research methods

### Type of study and settings

This was a qualitative study with health care teams in primary health care organisations from rural and urban areas. It was based on a grounded theory approach [21]. This study was part of a larger project “Scale-up an integrated care package for diabetes and hypertension for vulnerable people in Cambodia, Slovenia and Belgium” (SCUBY). SCUBY is an international research project funded by the EU under the Horizon2020 Framework Programme with contract number 825432 - SCUBY. Some of the results from the SCUBY project have already been published [22].

The study was performed in Slovenian primary care settings, in public community health centres (CHC). The health centres involved were CHC Ljubljana (several units), CHC Ravne na Koroškem, CHC Lendava and CHC Gornja Radgona.

The study of the overarching SCUBY project has been approved by the National Ethics Committee of Slovenia (ref: 0120–219/2019/4).

### Participants

Five to ten key informants participated in each focus group (FG) in each selected primary health care organisation (Table 1). The health care teams in Slovenia are multidisciplinary teams of health professionals with different profiles: FP, a practise nurse, a registered nurse, a registered nurse working in a health education centre, and a community nurse. We wanted to ensure that each focus group included at least one participant from a specific professional profile.

Practise nurse is a nurse with a bachelor’s degree who works with a FP [16]. A registered nurse is a nurse with a bachelor’s degree who works with a physician and is involved in treating patients for early detection of chronic diseases, providing health education, and collaborating with other professionals in caring for patients with chronic diseases [23]. She has a broad range of skills to meet the needs of all patients with multimorbidity [24]. A registered nurse working in a health education centre provides health education and self-management support [18]. A community nurse cares for patients at home when they are unable to see a physician [25].

**Table 1:** Characteristics of participants

Characteristics	N	%
Gender		
Male	4	8.3
Female	44	91.7
Age (years)		
< 30	5	10.4
30–39	16	33.3
40–50	13	27.1
> 50	14	29.2
Profession		
Family physician	11	22.9
Practise nurse	1	2.1
Registered nurse	20	41.7
Community nurse	10	20.8
Registered nurse working in a health education centre	6	12.5
Area of residence		
Urban	16	33.3
Rural	32	66.7
Area of doctor’s office		
Urban	26	54.2
Rural	22	45.8

### Instruments

The FG planned to solicit additional information from health professionals. These questions (Appendix 1) provide the general topic guide for the focus group discussions with questions listed for health care professionals/teams. The main topics discussed at FG are based on the implementation of the Integrated Care Package (ICP) as it is currently implemented in the organisation: Identification, Primary Care Treatment, Health Education, Self-Management, and Provider Collaboration [26]. In addition, questions about costs from the provider perspective and costs and barriers from the patient perspective were discussed.

General demographic questions about gender, age, living and working environments were collected to understand the diversity of respondents in the group.

### Data collection

Data collection took place from May to September 2019. Qualitative analysis was conducted by two independent researchers. FG conversations were recorded, for which participants signed an informed consent form. The researchers asked a question and the participants discussed the question and each participant expressed their opinion.

Participation in the study was voluntary, and participants signed an informed consent form to participate. They had the right to withdraw their consent at any time without having to justify the withdrawal.

### Data analysis

**Fig. 1:**
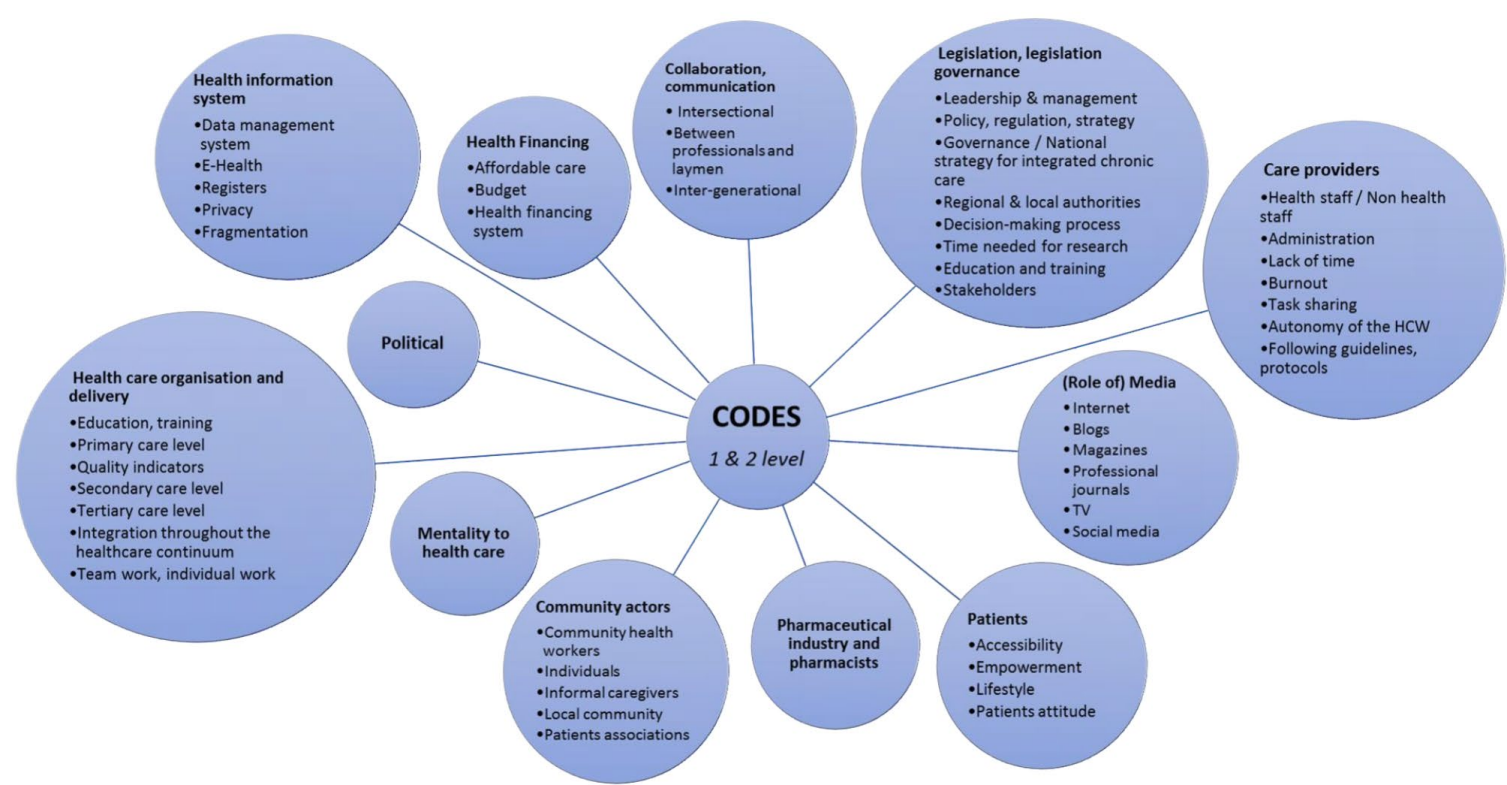
Level 1 and 2 codes

First, the FG discussions were transcribed verbatim. Then, the transcripts were coded using QSR NVivo 12 software. Inductive thematic analysis was performed. All transcripts were read by the researchers (NS, MML, ČZ) to obtain a more complete picture. As a result, codes and a coding tree were developed. As the analysis progressed, new codes and themes emerged (deductive approach) and finally a coding tree was developed with codes at the fourth or fifth level. The first level codes are the core themes that were identified and they generally allow for coverage of all issues that emerged during the coding process. They address and highlight both facilitators and barriers. Each additional level of coding is more specific and addresses a particular issue (Fig. 1).

For the coding process in the NVivo program, we imported all collected data into the program. FGs were coded by three team members. The fourth coder performed the review and analysis of the coded material. After coding, we proceeded with frame analysis. For this purpose, we sorted the coded material into a table in which we performed open coding. Each FG was assigned an interview code, and each FG quotes its number. The table itself contained the FG code and number, the coded quote, the concept, a subcategory, a category, and a theme. After the open coding was completed, we moved to axial coding of the data. This means that we sorted the coded material from the table in a hierarchical order, from general (a theme) to specific (the concept). Then we explained the analysed data separately in a descriptive manner. Data saturation was achieved.

### Trustworthiness of the results

Trustworthiness of the qualitative studies is ensured through five criteria: credibility, transferability, dependability, confirmability, and reflexivity [27]. To ensure credibility, we used investigator and data triangulation. To ensure transferability, we described not only the experiences of the participants, but also the context. To ensure dependability and confirmability, we used the audit trail approach, as we developed a clear research plan and adhere to it during all steps of the study.

## Results

Eight health care teams participated, five from an urban health centre (central Slovenia) and three from three rural health centres (two from the northern region and one from the eastern region of Slovenia). A total of 48 health professionals were involved. Each FG lasted one hour.

When analysing the results, we came across 12 main themes that emerged. Each theme has codes and subcodes that further define the theme. The major themes are listed below.

### Leadership, legislation, governance

As for the legislation, the respondents think that all the changes and improvements are too slow. They believe that health professionals should be trained regularly (e.g., in computer skills). At the formal level, they consider additional training (e.g., on diabetic foot) is important, and at the informal level, it is important to link health professionals with associations (e.g., retired nurses working in associations, training of lay consultants, training of health professionals) and to give them unhindered access to schools and the school system. In the area of long-term care, they emphasized the need to improve local treatment (e.g., strengthening home care services).

*“The system is such that 10 years pass before anything moves. Even if there are good ideas. I do not think anything is in a hurry, but everything is long-term.“ (TFSC 590)*.

*“Yes, the school system should be regulated to open the door to health care facilities. We have a problem with that. If the headmaster is for it, then it is not a problem, but if he is not for it, we have the door closed. “(TFSB 570)*.

### Health financing

The cost incurred by the patient may be in the form of a co-payment (test strips, certain medications, and health care services) or full payment (food, self-paid exams, blood pressure monitor, physical activity, and transportation). They said that healthy foods are very expensive and that there are financial constraints, especially for vulnerable groups. In addition, more and more medications have to be paid for. They pointed out the problem of patients with lower economic status, who often do not purchase additional health insurance. They also pointed out the problem of rural areas, where the economic situation is even worse than in the cities, people have lower pensions, the area is economically underdeveloped, and transport access is worse.

*“Let’s say for patients, the poorer economic situation is a problem because they often do not have supplementary insurance. And then, because they do not have supplementary insurance, certain medicines are subject to a fee.“ (TFSB 169)*.

### Health care organisation and delivery

In the context of health care organisation, primary, secondary, and tertiary levels of care, education and training, integration across the health care continuum, teamwork/individual work, and quality indicators were discussed. Health care professionals expressed that the current system of integrated care provides good accessibility and a method of diagnostic screening with integrated preventive examinations. Registered nurses point to the lack of information transfer between levels. They believe that more information about care (especially for relatives of the elderly) is needed, as this would reduce the burden on the health system (relatives would no longer go to the health centre for information). Many patients are referred to a health education centre for individual or group workshops or to a diabetologist at the secondary level. They may be referred to a diabetologist through a referral from FP. They said the collaboration between experts was good. They saw a particular advantage in the involvement of registered and community nurses.

*“Before the introduction of family medicine practices, we overlooked a large proportion of these patients. They just didn’t show up for checkups and we didn’t call them because of lack of time; now the registered nurse calls them herself and we have some control.“ (TFSC 378)*.

*“In the health education centre, we receive referrals when they are screened in the family medicine practice, all those who have high blood pressure or high sugar, to then enrol them in workshops.“ (TFSB 213)*.

*“We work very well with family medicine practices. If there is a patient who needs faster treatment or a service, we organize that.“ (TFSB 414)*.

*“The community nurse is ideally designed and of great importance here, the whole service. I think we are not sufficiently aware of the reach it has and the workload.“ (TFSA 265)*.

### Health workforce / care providers

Most health professionals referred to lack of time and too much administrative work. They said that health professionals are overworked because there are not enough staff, high demands, and too many patients. They said that patients often do not come and do not respond to invitations, especially younger patients.

“*On the negative side, I can point out that there is a lot of duplication, because we have to write cardboard plus computer and this computer, at least for me, takes a lot of time …and this administrative part…„ (TFSC 614)*.

*“It is not a problem to talk to the patient, it is not a problem to do the measurements. The problem is that you have to enter everything twice and three times… I would give the administration away…” (TFSB 362)*.

*“I think it would be nice if the doctor also had less registered patients, that there would be a smaller quantity.“ (TFSA 357)*.

*“We invite them, say, two or three times, and sometimes they call and say they do not want to come. The older ones are generally very responsive. The problem is the younger ones who do not respond to the invitation. The older ones like to come because they have a lot of problems.“ (TFSA 335)*.

### Patients

Patients have good access to measuring instruments and medical care. They have problems with transportation to health services because they do not have it, especially in rural areas. Patients behave quite irresponsibly because they do not follow the prescribed diet and do not take their medications regularly (some because of too many different medications and negative side effects). Many of them do not attend referral clinics and take poor care of their health because they lack their own motivation to lead a healthy lifestyle. They also say that patients who have never faced the disease in their lives have difficulty accepting it.

*“It seems to me that we are at a point where supply currently exceeds demand. Accessibility is complete. It is now up to the individual to respond to the invitation.“ (TFSC 131)*.

### Community actors / community link

They said that there are patient associations in Slovenia and that the diabetic association is spread all over the country. They offer support to patients and a space where they can educate themselves, socialise, offer support and share their own experiences.

They believe that patients listen to patients as teachers (lay consultants) who draw from their own problems and experiences. In this context, they suggest including lay consultants in the training groups in addition to experts.

*“That would be great. Because maybe other people would take them more seriously because they come from their own experience, and if they have a good handle on their illness, they can be wonderful teachers.” (TFSC 72)*.

### Collaboration/communication

Cross-sector collaboration is active primarily at the horizontal level. Experts collaborate by sharing health data and patient reports (e.g., the Centre for Health Education provides a report on patient success in the workshop). However, there is a need for recruitment and collaboration with dieticians, collaboration between the family medicine practice and the nursing home (e.g., report on patient data), and data sharing between experts on patient medication.

*“As for the treatment of diabetics in nursing homes, there is certainly much lacking here. There is much lack of integrated treatment on arrival at the nursing home. I think a report from the family medicine practice where the patient was cared for would be very welcome. On the medical certificate for admission to the home, which contains all his data, there is also this part which should be completed by the registered nurse. I have fought for this all my years in practice and despite hard battles and also working with the community nurse to get this done it just always comes up empty.“ (TFSG 434)*.

Collaboration would also contribute to better patient coverage and a more controlled environment. The problem also lies in the timeliness of information flow, as the patient waits for the results, which can lead to duplicate management.

### (Role of) media

The health care team said that the media does not give enough publicity to the family medicine practice and health education centre. They pointed out that the media contains too many harmful ads to promote an unhealthy lifestyle. They suggested promoting healthy lifestyles nationally and providing reliable information. They considered websites to be an important information tool because patients trust them. Television is mainly used for advertising, but it should contain more educational content. The problem with television is that each patient receives different information or interprets it differently.

*“Everyone has different information. Someone has heard something from television and has such information, someone has heard from some educational institutions and has other information, and it seems to me that … they come with very different information. (TFSB 185)*

### Pharmaceutical industry and pharmacists

In the pharmaceutical industry, they saw their role as educating and counselling patients about medications. They felt that the pharmaceutical industry was becoming more market-oriented and that patient counselling was not a priority.

*“Pharmacies should help more actively. I mean, they do not have to hire a master of pharmacy, which is expensive. There are many pharmaceutical technicians in the market who do not have a job, let them be consultants. Sometimes people just need simple advice.“ (TFSD 75)*.

### Health information system(s)

On this topic, respondents indicated that information transparency is poor and that there is no flow of information between levels. Due to the protection of personal data, the registered nurse in the family medicine practice does not have access to the data contained in the electronic data exchange platform with all the clinical reports of a single patient, so she does not have data on the patient’s results and the specialist does not see the community nurse’s report. They suggested allowing access to the data only for a valid reason and tracking who accesses the data. Administration is also time-consuming, as their work is duplicated by manually entering the personal card and entries into the computer.

*“The report is published via the “e-spine” program, but the registered nurse in the family medicine practice for patients she is supposed to look after once a year does not have this insight. And let’s assume that a patient who was at a diabetes specialist yesterday was invited to check up tomorrow, he will not get the mail home in two days, so he will not have the medical report with him … And you have no information.“ (TFSB 606)*.

### Mentality to health care

Respondents said that some patients feel that the health care system is responsible for them and that they should be taken care of. However, they themselves felt that patients should be given more responsibility. They also said that many patients do not take the disease seriously and many are irresponsible because they do not pick up their medications at the pharmacy and do not take care of a healthy lifestyle (they do not eat healthy, do not exercise, and do not follow medical advice).

*“ … They do not take it seriously enough … if they were aware of how much more power they have to influence their health with their lifestyle, that you do not just get a box of medicine … We are working in this area, but there are still huge reserves.“ (TFSB 15)*.

### Political

The health care team did not comment much on this issue, and similar issues are addressed in related legislative topics. However, they mentioned that in some cases there is a lack of cross-cutting collaboration and integration with social services, and responsibilities are not always clearly defined.

*“As well as politics… I can see that social welfare is very much interested in being involved and included. They have a very big appetite and are very strong politically. But in nursing, however, it does not move, does not allow them access, and then there is stagnation.“ (TFSA 513)*.

## Discussion

This study provided important insights into the micro-level perspectives of FP, nurses, and other members of the health care team who together form a whole and provide integrated care to the patient. It was found that health care professionals highlighted good accessibility and the method of diagnostic screening combined with preventive examinations as positive aspects of the current system of integrated care in Slovenia. They mentioned the good cooperation within the team, with the involvement of registered nurses and community nurses being a particular advantage. They complained about the high workload and the lack of manpower. They believe that patients do not take the disease seriously enough and that patients as teachers could be useful as educators.

Good interprofessional collaboration is necessary for comprehensive, high-quality, patient-cantered health care [28, 29]. Both in our study and abroad [30], they pointed out the importance of good collaboration within a team and saw a particular advantage in the inclusion of registered nurses and community nurses, who are a kind of link between the patients and the physician. They emphasized the importance of the role of registered nurses and that they contribute greatly to patient care, whereas in the UK, the role of nurse practitioners/registered nurses in general practice is viewed with great concern by FP. They feel threatened in terms of their status, professional and financial security, and nursing skills, and they see barriers in the structure and organization of the work [31]. According to international data, the role of the primary care nurse has expanded because of the shortage of FP and the growing burden of chronic disease and multimorbidity [32]. In Belgium, although registered (practice) nurses, physicians, and patients agreed that the involvement of a nurse practitioner in primary care had a number of benefits, they also expressed concerns about responsibility, trust, and accountability, which hindered interprofessional teamwork [28]. In our study and in Switzerland [32], the opposite was found: FPs trusted the registered nurses and worked well with them. Their tasks are management of patients with stable chronic diseases, implementation of preventive measures (screening) leading to early detection of risk factors and selected chronic diseases [16, 19]. The basis for trust and good collaboration is clearly defined structures, team development, and shared visions of care and collaboration [28, 29]. In our health care system, a good structure is ensured because all family medicine practices in Slovenia treat chronic patients with a standardized approach using protocols [16]. The shortage of medical staff, especially FP and nurses, is not unique in our country. According to the data in the literature, the problem of providing human resources/adequate medical staff seems to be more widespread than we think [31, 33]. Therefore, we should strive to reduce unnecessary administrative burden, which was also highlighted in our study. In Slovenia, up to 25% of all visits to the family medicine practices are administrative in nature [34, 35].

Two important suggestions from our study were to involve other professionals in the management of chronic patients and to strengthen the role of lay people in patient education. Both were also voiced by WHO with an expanded Kaiser pyramid that places more emphasis on self-care and integrated care, and in the studies for low- and middle-income countries (LMICs) [2, 35–39]. The first steps in this direction have already been taken in Slovenia with the introduction of the registered nurse into the primary care team. This has proven to be effective [20, 25, 40]. However, patient education is also needed to reduce the burden on medical staff and to empower patients to take more responsibility for their own health.

In the context of discharge, other studies [26, 41, 42], such as ours, have shown that patients can act as educators (i.e., lay consultants or peer supporters) as part of a team working with professionals because patients can more easily identify with and trust them, as lay consultants speak from their own experience of managing a particular illness. They may also contribute to better clinical outcomes [43, 44]. Our study suggests that primary care teams would be willing to accept lay people as supporters in the management of the chronically ill.

Because most participants in our study reported a lack of information, there is a need for better information flow between levels of care and with certain other professionals or institutions (dietitians, collaboration between family medicine practice and nursing home), for example, by sharing data and allowing online meetings [22]. This would facilitate integration and improve integrated patient care. Collaboration and communication between the family medicine practices and other professional settings should be strengthened and the flow of information improved.

Successful implementation and improvement of integrated care requires collaborative approaches that use an iterative cycle of research, action, and reflection. However, it should be recognized that these processes of collaboration, co-creation, learning, and reflection take time [45]. As history shows, reorienting health services is a slow process that requires political commitment and listening. Each country can set its own goals for integrated and people-centered health services and develop its own strategies to achieve them [3].

### Strengths and limitations of the study

The strength of this study is that different profiles of primary-level health care teams participated in the study and expressed their views on integrated care for patients with hypertension and type 2 diabetes in primary care and identified barriers and facilitators to scaling up care. With the help of providers at different levels, we can achieve better outcomes in integrated care and improve people’s experience of care.

A limitation of this study is that it was performed in only a part of the total health centres in Slovenia. With the selection, we tried to cover all regional differences, but the FGs of urban organisations were from the same region and there were only three rural organisations from two rural regions. Therefore, we might have overlooked some important data. However, we tried to assure trustworthiness of the results so we believe that the barriers and facilitators we found among FGs in this context are likely to be found among other FGs throughout the country.

## Conclusion

Primary care teams described the importance of implementing integrated care for diabetes and hypertension patients at four levels: patients, community, care providers, and state. Primary care teams also recognized the importance of adding more professionals from different health care disciplines to their team. These findings can help other countries to develop plans for integrated care implementation in their primary care.

Further qualitative research with health care teams and others involved in integrated care (e.g., lay consultants) would be valuable to explore these ideas further.

## Electronic supplementary material

Below is the link to the electronic supplementary material.

Supplementary Material 1

## Data Availability

The datasets generated and/or analysed during the current study are available from the corresponding author on reasonable request.

## List of abbreviations

FG: Focus group
FP: Family physician
ICP: Integrated Care Package
NCDs: Noncommunicable Diseases
SCUBY: Scale-up an integrated care package for diabetes and hypertension for vulnerable people in Cambodia, Slovenia and Belgium
WHO: World Health Organization
