# Supplementary material for: Perceptions of the primary health care team about the implementation of integrated care of patients with type 2 diabetes and hypertension in Slovenia: qualitative study
Source: BMC Health Serv Res. 2023 Apr 12;23:362. doi: 10.1186/s12913-023-09353-3 (PMC10091568; doi:10.1186/s12913-023-09353-3)
Supplement: Supplementary file 1 — Supplementary Material 1 [file 12913_2023_9353_MOESM1_ESM.docx]

Appendix 1. Thematic guide for focus groups.

| **Introduction** |
| --- |
| **Introduction of the researchers:**   - Thank - Name and function of researchers   **Introduction of SCUBY:**   - SCUBY is a four-year research project on the scaling up of integrated care for type-2-diabetes and hypertension. We are studying the scaling-up of integrated care for type-2-diabetes and hypertension, with special attention to vulnerable people. - The aim of this research is to engage with stakeholders at all levels, including patients and healthcare staff, to identify opportunities and barriers to integrated chronic care and to support and implement best practices on a larger scale. - Key terms: integrated chronic care, diabetes, hypertension and vulnerable groups   **Explain purpose and the intent of the interview:**   - Aim of interview: gaining insight into opinions and perceptions of patients, health care teams and community actors in relation to integrated care, its barriers and facilitators - Duration of focus group interview (up to 90 minutes) - Ensure confidentiality - Questions?   **Informed consent:**   - Ask (to sign) the informed consent and permission to record the interview |
| **A. Questions to doctors/health care staff/teams** |
| **Opinion on current process:**   - Which adaptations were made at the implementation of the integrated care package at the health care facility and at other organisations involved? - Which external organisations were involved to support implementation? - How were health workers, other actors and patients prepared for implementation? - What was the role of local and central health authorities in the implementation?   **Facilitators:**   - In your opinion, what is good in the current implementation of the integrated care for people with type-2-diabetes and hypertension? - How are vulnerable population identified? How well does ICP reach vulnerable people? - What are important factors that facilitate the current way of working? What are further options for improving management (detection, treatment, and self- management) of people with type-2-diabetes and/or hypertension? Especially regarding vulnerable people? - How do you see the directions of possible extension of care/scaling-up and facilitators to that? - Who are potential people/approaches that can be added to the existing care? - How do you see the role of patients as teachers? How do you see the role of organisations in the community? How do you see the role of informal caregivers?   **Barriers/obstacles:**   - What are your personal experiences/what kind of obstacles (in screening, testing, treatment) have you considered in the ICP-process (screening, testing, diagnosing, retaining in care, follow-up, self-management, different levels cooperation) for (vulnerable) patients with type-2-diabetes and/or hypertension at the primary level? - What are the problems of people who don’t come regularly for care? What are current actions you take? What are other options? - Where are the obstacles that would prevent expansion or change in the process?   **Care and financial barriers:**   - What according to you are major financial barriers to appropriate health care for patients with type-2-diabetes and/or hypertension treatment? |
